# Meta-analysis of clinical adverse events after CABG vs. PCI in patients with chronic kidney disease and coronary artery disease

**DOI:** 10.1186/s12872-023-03560-w

**Published:** 2023-11-30

**Authors:** Cheng Luo, Qiang wang, Shuxiong Nong, Yushan Chen, Longchang Li, Chun Gui

**Affiliations:** 1 Department of Cardiology, The First Affiliated Hospital of Guangxi Medical University, Nanning, Guangxi Zhuang Autonomous Region 530021 China; 2 Department of Cardiology, The Fourth Affiliated Hospital of Guangxi Medical University, Liuzhou, China; 3 Guangxi Key Laboratory Base of Precision Medicine in Cardiocerebrovascular Diseases Control and Prevention, Guangxi Clinical Research Center for Cardiocerebrovascular Diseases, Nanning, Guangxi Zhuang Autonomous Region 530021 China

**Keywords:** Clinical adverse events, Cardiovascular Disease, meta-analysis, Chronic Kidney Disease

## Abstract

**Aim:**

To investigate the efficacy and postoperative clinical adverse events of coronary artery bypass grafting (CABG) or percutaneous coronary intervention (PCI) for chronic kidney disease (CKD) study participants combined with coronary artery disease (CAD).

**Methods:**

All randomized controlled trials (RCTs) that focus on the therapeutic effect evaluation of CABG and PCI and their effect on postoperative clinical adverse events as well as main adverse cardiovascular and cerebrovascular events (MACCEs) in CKD study participants with CAD were screened from the following databases, including CNKI, CBM, Wan Fang, VIP, Embase, PubMed, as well as Cochrane library clinical controlled trials. The study was conducted under the PRISMA 2020 criteria. Data were extracted, and quality control was evaluated from the modified Jadad rating scale. Meta-analysis was then undertaken through STATA 16.0 software.

**Results:**

A total of 5 RCTs were obtained, including 1198 patients. Study participants were subdivided into two groups, including the PCI group (n = 604) and the CABG group (n = 594). Meta-analysis of clinical adverse events results showed that the long-term survival results of CAD patients with CKD who underwent PCI were worsened compared to CABG, such as long-term MACCEs (RR = 1.59, 95%CI: 1.04–2.43) and the long-term repeated revascularization (RR = 2.48, 95%CI: 1.76–3.49). Also, cardiac death (RR = 1.68, 95%CI:1.04–2.71), as well as cerebrovascular accident (RR = 1.74, 95%CI:1.04–2.90) in CABG group was significantly lower than that in PCI group.

**Conclusion:**

This meta-analysis showed that CABG provided a better therapeutic effect than PCI in CKD patients with CAD when considering long-term prognosis. However, more prospective RCTs are needed to define the proper revascularization strategy for CAD patients with CKD.

## Introduction

Chronic kidney disease (CKD) generally has a worse prognosis after invasive procedures such as PCI or CABG than patients with healthy renal function. Coronary artery disease (CAD) is the main factor of death induced by cardiovascular events in CKD study participation [1]. CAD patients with CKD have serious and complex coronary artery lesions, resulting in a poor prognosis and a large economic burden on patients [2]. A study conducted by Baber et al. [3] concluded that the risk of mortality, major adverse cardiovascular and cerebrovascular events (MACCEs), myocardial infarction (MI), as well as stroke is significantly higher in study participants with CKD compared with those without CKD. In a study of left main artery remodeling by Giustino et al. [4], mortality and incidences of major adverse cardiovascular events were significantly higher in CAD study participants with CKD than in CAD patients without CKD. Therefore, we should pay more attention to how to find more effective cardiac treatment strategies for these patients.

Recent studies have revealed that for CAD patients with CKD, timely diagnosis and early PCI or CABG have a lower mortality rate than drug therapy [5, 6]. Previous research has discovered that although CABG increases the incidence of short-term acute kidney injury, patients who underwent CABG have more favorable survival outcomes than those who underwent PCI [7, 8]. However, Kang et al. [9] conducted follow-up research including 2,108 CKD study participants with multi-vessel CAD, which concluded PCI utilizing drug-eluting stents had similar composite outcomes for stroke, all-cause death, or myocardial infarction (MI) compared with CABG group.

The 2018 European Society of Cardiology/ European Association for Cardio-Thoracic Surgery (ESC/EACTS) Guidelines recommend CABG over PCI in patients with moderate to severe CKD with multi-vessel disease [10]. Similarly, the American College of Cardiology/American Heart Association (ACC/AHA) guidelines recommend that CABG is superior to PCI in patients with end-stage renal disease (ESRD) accompanied by three-vessel disease, proximal left anterior descending artery (LAD) disease, plus other major artery disease [11]. However, these guidelines recommend mostly based on observational studies comparing bypass surgery with first-generation drug-coated stents or bare metal stents [12]. Results of an observational study by Bangalore et al. [13] show that in patients with CKD who underwent coronary revascularization, bioabsorbable polymer-coated platinum chromium everolimus-eluting stent (BP-EES), compared to those who underwent CABG surgery, were less frequent need for repeated revascularization and of significantly lower risk of death and stroke at one month. Thus, there is still a controversy as to whether CABG or PCI is better for patients with CKD combined with CAD. Therefore, we implemented a meta-analysis of randomized controlled trials (RCTs) that had already been published to select a more appropriate treatment for CKD patients with CAD.

## Materials and methods

### Literature retrieval

The published studies using PCI and CABG outcome data in CKD study participants with CAD were systematically reviewed in accordance with the Preferred Reporting Project (PRISMA) Guidelines for Systematic Review and Meta-analysis [14]. As of February 10, 2023, we have conducted searches of studies related to this topic on the following databases, including PubMed, The Cochrane Central Register of Controlled Trials, Embase Database, Chinese Journal Full-text Database, Chinese Biomedical Literature Database, Wanfang Database, as well as Chinese Sci-tech Journal. The retrieval search strategy is “chronic kidney disease AND coronary artery bypass grafting AND percutaneous coronary intervention”.

### Eligibility criteria for inclusion and exclusion

The studies where quantitative raw data can be obtained or the risk ratio (RR) can be calculated were included. If the study patient groups overlap, select a study with a larger sample size. Studies that meet the following criteria are included: [1] Comparison of CABG and PCI data [2]. The patients participating in the study have CKD (eGFR < 60 ml/min/1.73 m² or Ccr < 60 mL/min) [3]. Randomized Controlled Trial (RCT). Research with the following conditions will be excluded: [1] Repeated research [2]. Meta-analysis, review, meeting summary, or agreement [3]. The postoperative results data on the comparison between CABG and PCI was not available. The main results of concern are all-cause death, MACCEs, cardiac death, MI, the need for revascularization, and cerebrovascular accidents. Long-term follow-up refers to a follow-up period greater than three years. All studies were reviewed by two authors independently. The two authors evaluated the quality of the included studies as well as extracted relevant data. Our third author would assist in the settlement based on the standard if there were disagreements.

### Evaluation of data quality and extraction of data

Standardized data tables were utilized to extract information. The following data related to the study, patients, and outcomes were extracted: author, year of publication, design of experiment, sample in time, sample size, age, gender, average follow-up period, eGFR value, smoking, diabetes, hypertension, hyperlipidemia, history of myocardial infarction as well as unstable angina history, history of peripheral vascular disease, myocardial involvement lesion blood vessel, main not conscience for all-cause death, MACCEs, cardiogenic death, MI, repeated revascularization, as well as cerebrovascular accident. The RoB2 scale was utilized to evaluate the quality of the study by two independent authors [15].

### Definition and outcome metrics

In CKD, the diagnostic standard was estimated as glomerular filtration rate (eGFR) < 60 mL/min/1.73 m^2^. CAD is defined as the presence of stenosis of ≥ 50% in the left main or ≥ 70% in any other epicardial coronary artery. The MI definition follows the 4th Universal definition of MI, like most papers currently [16]. Outcome measures were as follows: long-term all-cause and short-term all-cause death, MACCEs, sudden cardiogenic death, MI, repeated revascularization, as well as cerebrovascular accident. Due to the material heterogeneity of the results reported in the studies, results were based on the following pre-specified definitions. Long-term all-cause death refers to deaths that occur throughout > 3 years. Short-term all-cause deaths were referred to as all-cause deaths within 30 days. A MACCE refers to a cardiovascular composite endpoint event that includes all-cause death, non-fatal MI, cerebrovascular accident, as well as repeated revascularization. The term MI refers to the significant change in biomarkers for damaged cardiac tissue in conjunction with obvious signs or symptoms of patients that indicate disease related to cardiac ischemia. The cerebrovascular accident was diagnosed by a neurologist as neurological deficits based on imaging, such as stroke events, transient ischemic attacks (TIA), as well as reversible ischemic neurological deficits.

### Analysis of statistical data

Statistical analysis was conducted using STATA 16.0 software. RR as well as the corresponding 95% confidence interval (CI) are analyzed. Statistically significant was defined as *P* < 0.05. The I² statistic was utilized to check the heterogeneity of the study. I^2^ values of 25–50%, 50–75%, as well as > 75%, were low, medium as well as high heterogeneity [17], respectively. The fixed-effects model is utilized if I^2^ < 50% and the random-effects model is utilized in the statistical analysis if I^2^ > 50%. When heterogeneity was high, sensitivity analysis was implemented after the exclusion of studies one by one. In order to evaluate the latent impact of publication bias, funnel plot asymmetry tests were conducted.

## Results

### Baseline characteristics of patients

**Fig. 1:**
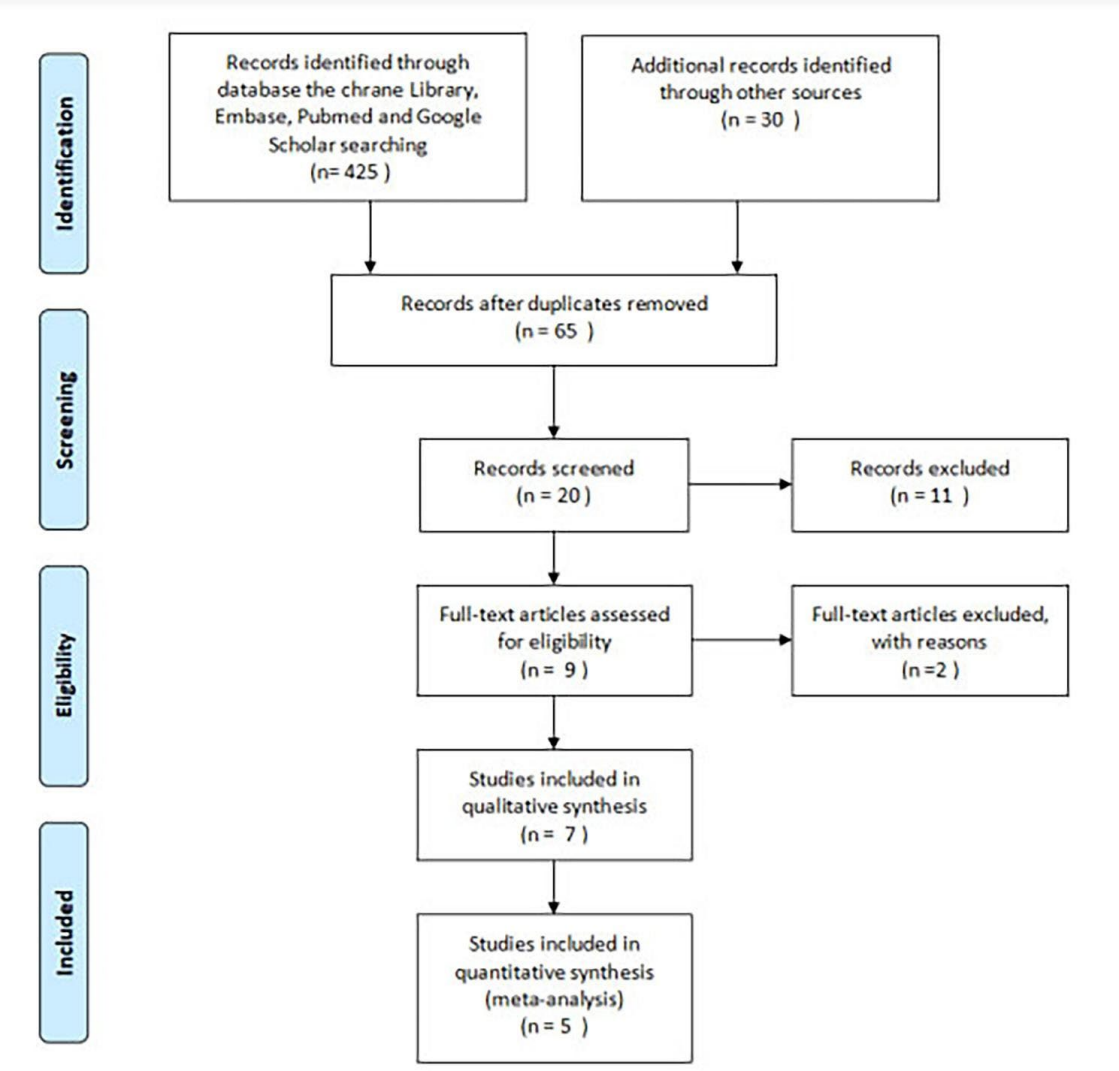
Literature screening diagram

Our pre-designated literature search identified 465 articles. After reviewing the title and abstract, 65 citations were reviewed in detail. However, we excluded 55 studies due to the inconformity of the designated inclusion criteria. A total of 5 trials were eligible for inclusion (Fig. 1). All the studies used randomized processing results analysis. A total of 1198 randomized subjects (PCI: 604; CABG: 594) were included, and the follow-up time after the intervention was 3–10 years. The baseline characteristics of the enrolled study participants are represented in Tables 1 and 2. CKD patients tend to be older and the proportion of male patients is higher.

**Table 1:** Study characteristics and sample size, age, sex, follow-up time, outcome indexes, and eGFR evaluation methods (PCI vs. CABG).

Authors	Year	Country	patients(n)			Age (years ± SD)		Male (n, %)		Follow-up (Yrs.)	Outcome indexes	eGFR evaluation
			PCI	GABG	Total	PCI	GABG	PCI	GABG			
Lima et al. (18)	2020	Brazil	49	47	96	NR	NR	NR	NR	10	All-cause of death, MI	MDRD equation
Milojevic et al. (8)	2018	Multi-country	158	151	309	72 ± 7	72 ± 8	108 (68)	101 (67)	5	All-cause of death, MACCEs, Cardiac death, MI, Repeated revascularization, Cerebrovascular accident	CKD-EPI equation
Giustino et al. (4)	2018	USA	177	184	361	NR	NR	NR	NR	3	All-cause of death, MACCEs, Cardiac death, MI, Repeated revascularization, Cerebrovascular accident, In-stent thrombosis, Bleeding	CKD-EPI equation
Ix et al. (19)	2005	USA	151	139	290	68 ± 6	69 ± 7	86 (57)	78 (56)	3	All-cause of death, MI, Repeated revascularization, Cerebrovascular accident	Cockcroft-Gault formula
Aoki et al. (20)	2005	Multi-country	69	73	142	70 ± 6	71 ± 6	44 (64)	53 (73)	5	All-cause of death, MACCEs, Cardiac death, MI, Repeated revascularization, Cerebrovascular accident, Pathological Q wave	Cockcroft-Gault formula

MACCEs: Major cardiovascular and cerebrovascular adverse events, MI: myocardial infarction, eGFR: estimated glomerular filtration rate, MDRD: Modification of Diet in Renal Disease, CKD-EPI: CKD Epidemiology Collaboration, NP: not reported

**Table 2:** eGFR values, smoking rate, cardiovascular risk factors, and previous history and ejection fraction (PCI vs. CABG)

Authors	Year	Multi-vessel CAD	eGFR (ml/min/1.73 m²)	Invention		Smoke (n, %)		Diabetes (n, %)		Hypertension (n, %)		Hyperlipidemia (n, %)		History of MI (n, %)		History of unstable angina attacks (n, %)		History of peripheral vascular disease (n, %)		EF (%, mean ± SD)	
				PCI (n)	GABG (n)	PCI	GABG	PCI	GABG	PCI	GABG	PCI	GABG	PCI	GABG	PCI	GABG	PCI	GABG	PCI	GABG
Lima et al. (18)	2020	yes	< 60	49	47	NR	NR	NR	NR	NR	NR	NR	NR	NR	NR	NR	NR	NR	NR	NR	NR
Milojevic et al. (8)	2018	no	mean eGFR	47.6 ± 10.8	47.6 ± 10.8	15 (10)	18 (12)	44 (29)	50 (33)	135 (86)	128 (85)	122 (78)	119 (79)	58 (37)	47 (32)	42 (27)	44 (29)	21 (13)	24 (16)	NR	NR
Giustino et al. (4)	2018	no	< 60	177	184	NR	NR	NR	NR	NR	NR	NR	NR	NR	NR	NR	NR	NR	NR	NR	NR
Ix et al. (19)	2005	yes	< 60	151	139	26 (17)	13 (9)	32 (20)	22 (16)	71 (47)	75 (54)	82 (54)	79 (57)	59 (39)	56 (40)	47 (31)	43 (39)	8 (5)	11 (8)	60 ± 13	59 ± 13
Aoki et al. (20)	2005	yes	Ccr (< 60 mL/min)	69	73	11 (16)	8 (11)	15 (22)	11 (15)	36 (52)	34 (47)	40 (58)	38 (52)	NR	NR	22 (32)	26 (36)	4 (6)	5 (7)	61 ± 13	58 ± 13

CAD: coronary artery disease, Ccr: creatinine clearance rate, eGFR: estimated glomerular filtration rate, MI: myocardial infarction, EF: ejection fraction, NP: not reported

### Literature quality evaluation

**Fig. 2:**
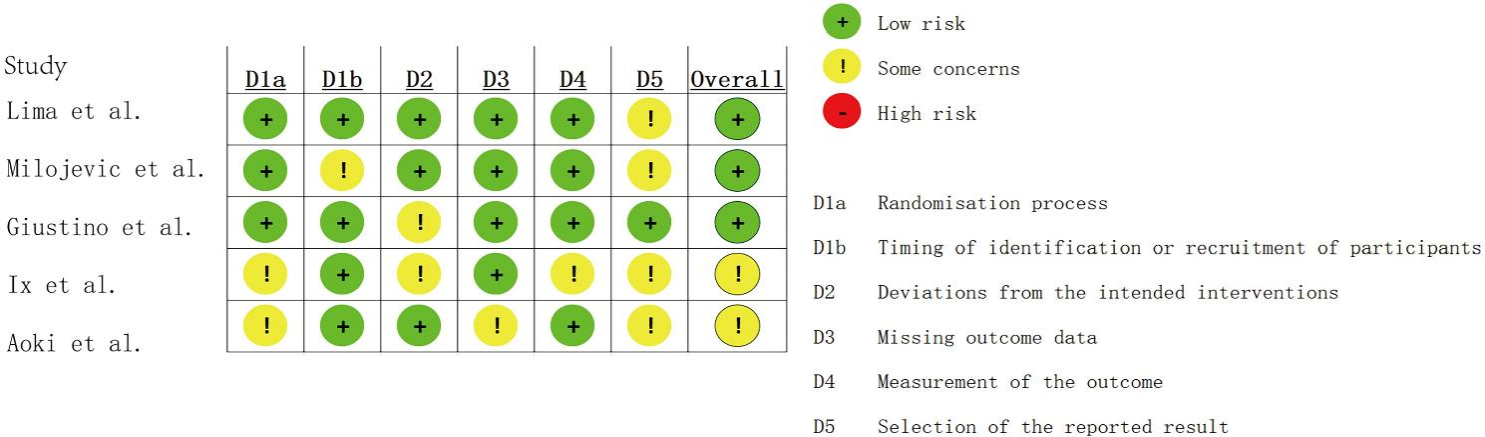
Quality assessment of studies via the Risk of Bias 2 (RoB 2) tool

The quality evaluation process of the five included studies was assessed in accordance with the modified Jadad scale. Finally, based on the evaluation results of 3 researchers, there were three high-quality and two low-quality articles. The quality evaluation form according to the RoB2 scale (the risk of bias was classified into three levels: “low risk of bias,“ “some concerns,“ and “high risk of bias.“) is shown in Fig. 2.

### All-cause death

**Fig. 3:**
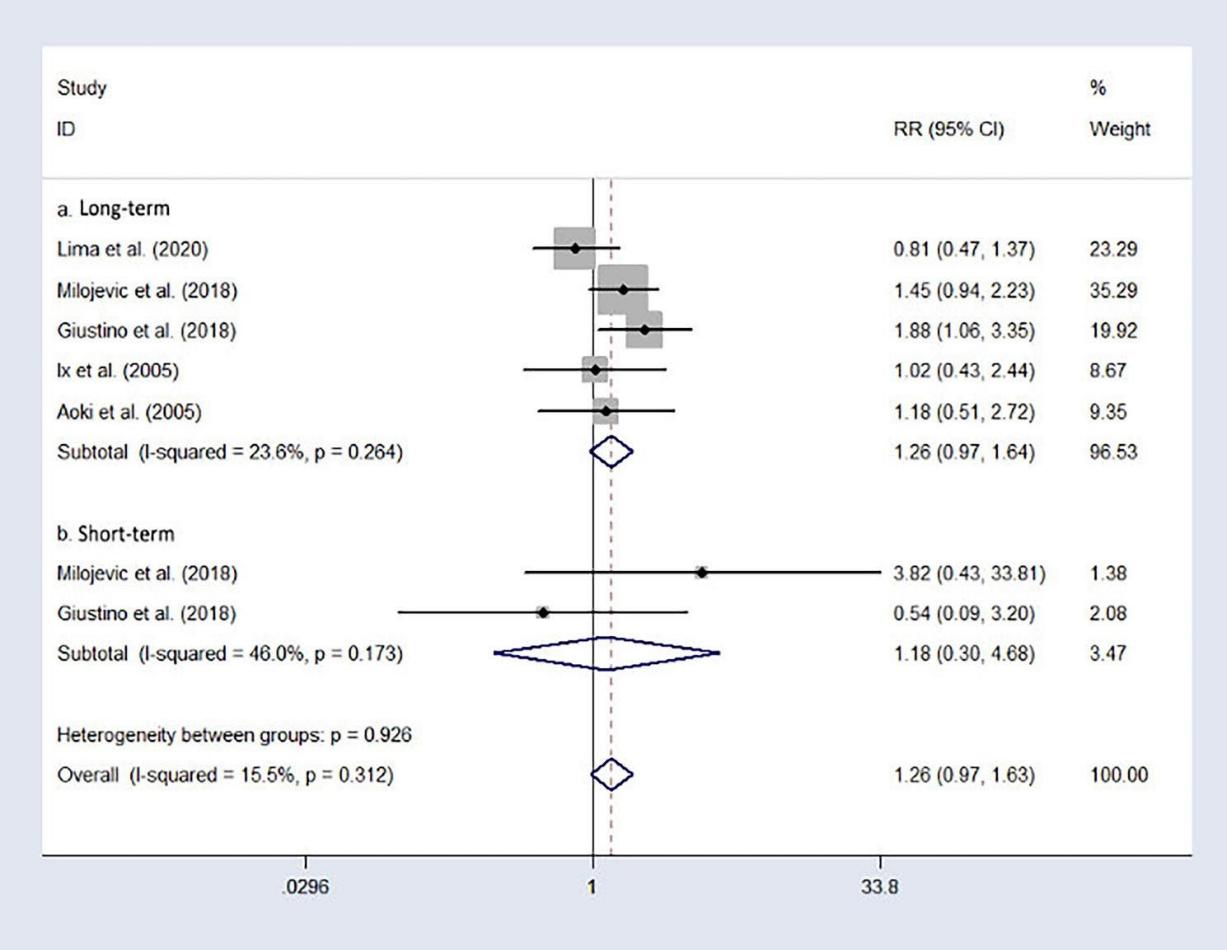
Forest plot of all-cause death after treatment (PCI vs. CABG).

Five research analyzed the long-term all-cause death events. The long-term mortality risk of participants who underwent CABG was slightly lower than that of the participants who underwent PCI (RR = 1.26, 95%CI: 0.97–1.64), without significant heterogeneity (I²=23.6%). The result is represented in Fig. 3. a. After the analysis of the subgroup was limited to short-term mortality risk, there were no significant differences in short-term mortality risk between the participants who underwent CABG and PCI (RR = 1.18, 95%CI: 0.30–4.86, I²=46.0%). The result is represented in Fig. 3. **b**.

### MACCEs

**Fig. 4:**
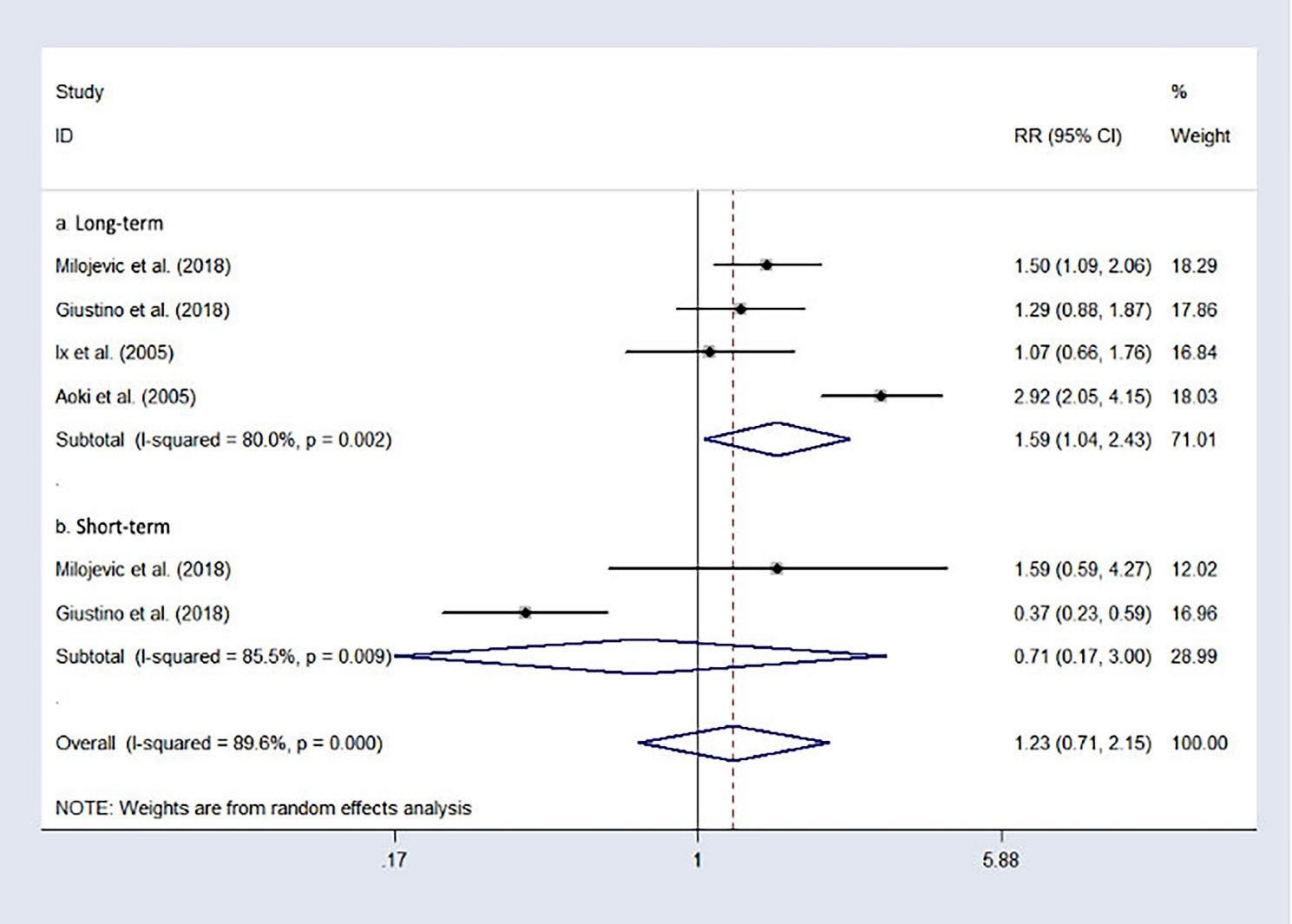
Forest plot of the major cardiovascular and cerebrovascular adverse events after treatment (PCI vs. CABG).

Four studies reported long-term MACCEs. Due to the high heterogeneity (I² = 80.0%, p = 0.002), the random-effects model was applied for analysis. The combined results show that the long-term risk of MACCEs in CABG is lower than that of PCI (RR = 1.59, 95%CI: 1.04–2.43), which is represented in Fig. 4. **a**. However, after the analysis of the subgroup was limited to the short-term risk of MACCEs, it showed different results. There were no significant differences in short-term risk of MACCEs between the participants who underwent CABG and PCI (RR = 0.71, 95%CI: 0.17-3.00, Fig. 4. **b**), with significant heterogeneity (I²=85.5%).

### Cardiac death

**Fig. 5:**
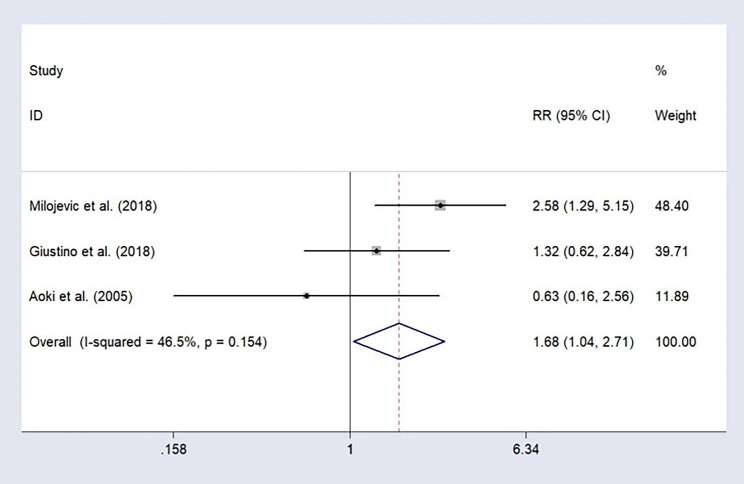
Forest plot of cardiac death after treatment (PCI vs. CABG).

Three studies reported long-term cardiac death. Based on the pooled analysis results of these three studies based on a fixed-effect model, the long-term cardiac mortality rate of patients receiving CABG was significantly lower than that of patients receiving PCI (RR = 1.68, 95%CI: 1.04–2.71; I^2^ = 46.5%; Fig. 5).

### MI

**Fig. 6:**
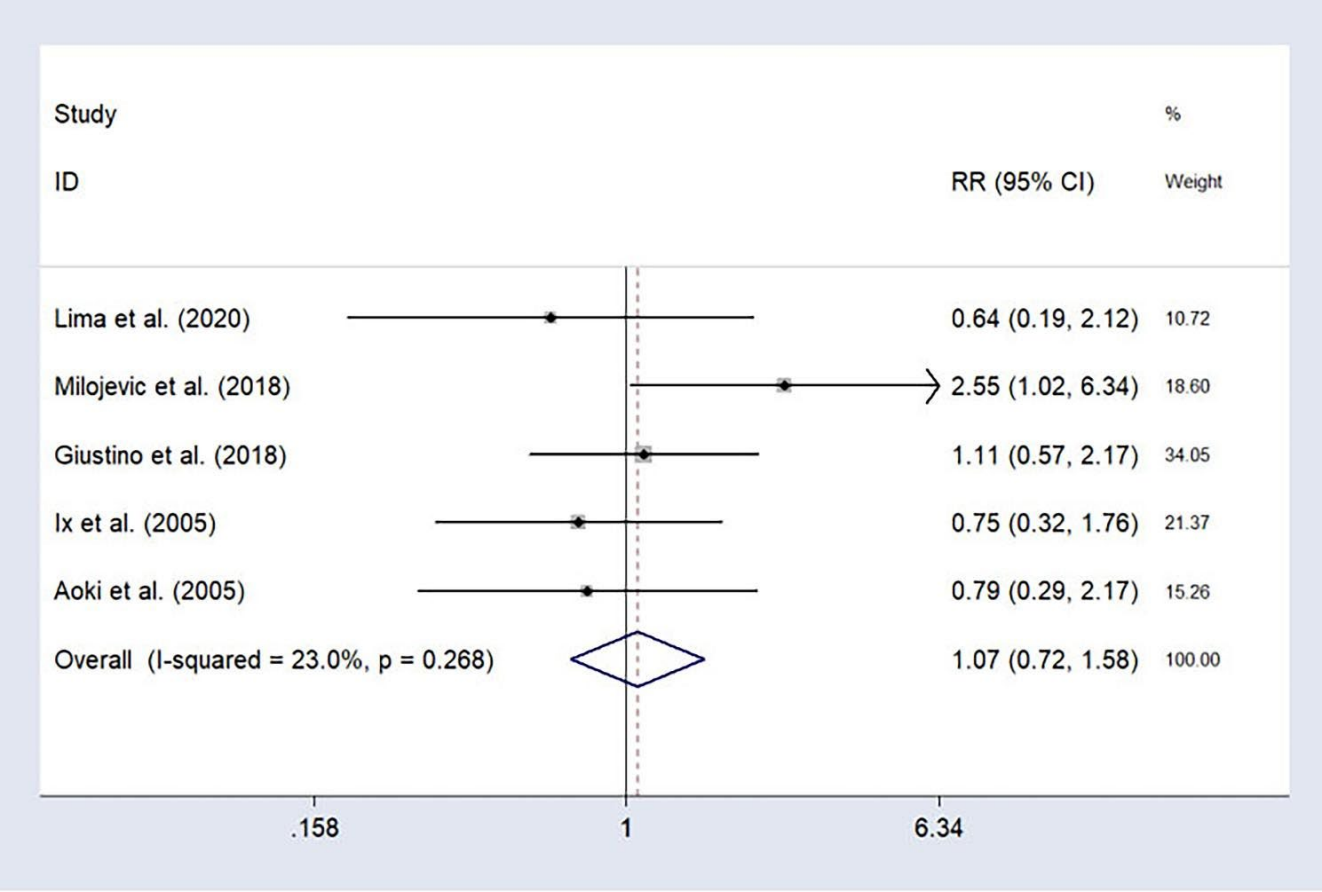
Forest plot of myocardial infarction after treatment (PCI vs. CABG).

Five included studies reported long-term myocardial infarction and the results showed low heterogeneity (I^2^ = 23.0%, P = 0.268); using a fixed-effect model, the results shown in Fig. 6: RR = 1.07, 95%CI: 0.72–1.58. It can be considered that there were no significant differences in long-term risk of myocardial infarction between the participants with who underwent CABG and PCI.

### Repeated revascularization

**Fig. 7:**
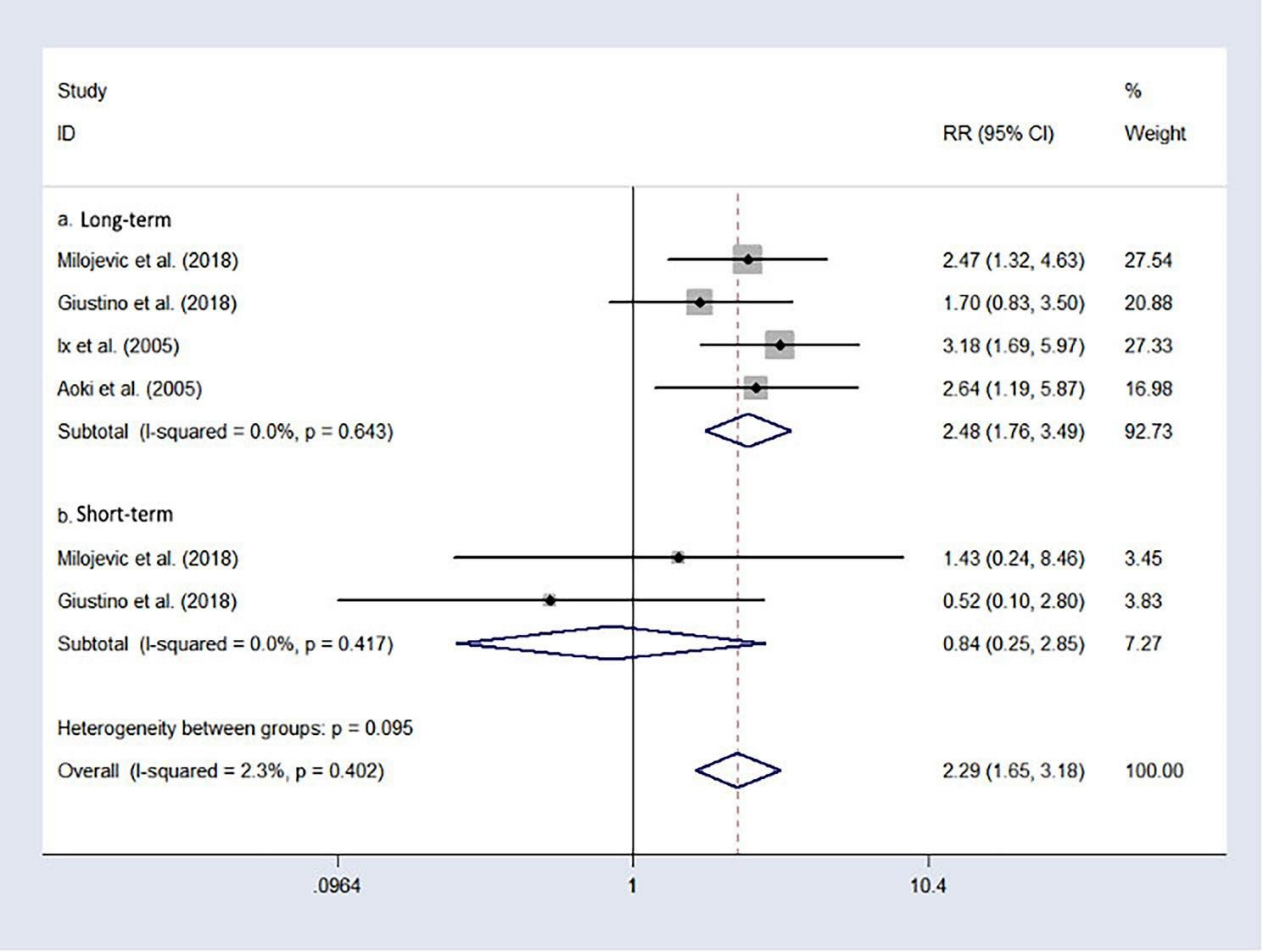
Forest plot of repeated revascularization after treatment (PCI vs. CABG).

Four studies reported repeated revascularization without heterogeneity (I^2^ = 0.0%, P = 0.643). Therefore, the fixed-effect model was utilized. The long-term risk of repeated revascularization of participants who underwent PCI was higher than that of participants who underwent CABG (RR = 2.48, 95%CI: 1.76–3.94, Fig. 7. **a**). When the subgroup analysis referred to the short-term risk of repeated revascularization, it showed different results. There were no significant differences in short-term risk of repeated revascularization between the participants with who underwent CABG and PCI (RR = 0.84, 95%CI: 0.25–2.85, Fig. 7. **b**), without heterogeneity (I^2^ = 0.0%).

### Cerebrovascular Accident

**Fig. 8:**
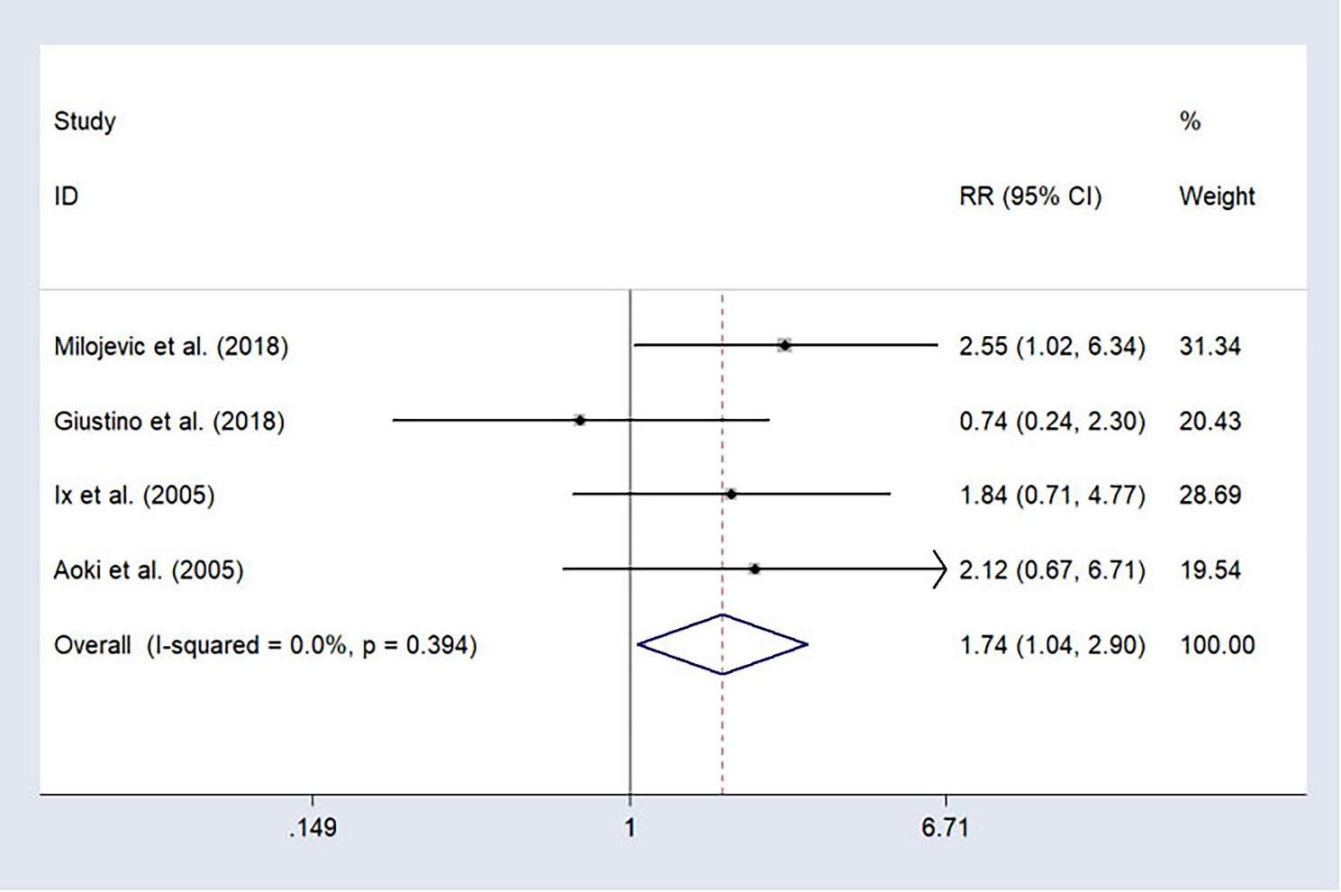
Forest plot of the cerebrovascular accident after treatment (PCI vs. CABG).

We pooled four research on cerebrovascular accidents, which represented no heterogeneity (I^2^ = 0%, p = 0.394); therefore, the fixed-effects model was utilized. The results indicated that the risk of long-term cerebrovascular accidents of participants who underwent PCI was significantly higher than that of participants who underwent CABG (RR = 1.74, 95%CI: 1.04–2.90, Fig. 8).

## Discussion

The purpose of our research is to conduct a meta-analysis to compare the long-term and short-term outcomes of CKD patients receiving invasive treatment, including CABG and PCI. Eventually, five relevant RCTs were published [4, 8, 18–20]. Our results show that CABG in CKD patients is associated with a lower risk of long-term MACCEs, cardiac death, long-term repeated revascularization, and cerebrovascular accident compared with PCI. However, the long-term recurrence rate of MI was similar in the participants who underwent CABG and PCI cohorts. Interestingly, there were no significant differences in short-term risk of all-cause death, MACCE and repeated revascularization among the participants with who underwent CABG and PCI.

A previous meta-analysis of 2 trials and 15 retrospective trials (including 62,343 CKD patients) reported that compared with PCI, CABG is associated with lower long-term mortality, MI, and repeated revascularization risk, and these results are consistent with our meta-analysis [21]. The heterogeneity of MACCEs’ RR in our results is significantly high, possibly resulting from the defect of the design of the experimental process as well as the different features in the eligibility criteria of inclusion and exclusion between research. PCI usually treats the culprit vascular disease that causes obvious symptoms. Nevertheless, other residual vascular stenoses will redevelop after undergoing PCI operation, inducing revascularization development to be incomplete. The growing risk of incomplete revascularization is regarded as the primary factor of adverse cardiovascular events, including MI, repeated revascularization, as well as cardiac death [22]. On the contrary, participants undergoing CABG can be provided with new blood vessels to substitute the culprit blood vessels, which ensures a greater possibility of achieving revascularization completely than participants undergoing PCI. Moreover, because of the requirement for repeated revascularization, CKD patients receiving PCI undergo routine coronary angiography follow-up more frequently than patients receiving CABG, and coronary angiography also increases the incidence of adverse events to a certain extent [23]. Recent research has revealed that the incidence of stroke in the participants who underwent CABG may be reduced because of the utilization of the off-pump surgery technique as well as the avoidable utilization of aortic clipping [13]. Even though the long-term events of participants undergoing PCI are not as favorable as participants undergoing CABG, PCI still has advantages over CABG, including a lower incidence of infection, shorter discharge time, and faster recovery.

Our study found that PCI was significantly less effective than CABG in terms of repeated revascularization and cardiovascular mortality endpoints. Several possible reasons can be analyzed: (1) CKD is often secondary to diabetes [24, 25], and diabetes can significantly increase the risk of in-stent restenosis in CAD patients after PCI, leading to a more frequent need for repeated revascularization. (2) CAD combined with CKD often manifests as diffuse multi-vessel lesions [26]. For these patients, PCI is generally difficult to achieve complete revascularization, with limited improvement in myocardial ischemia and increased risk of angina recurrence. (3) CKD patients often have severe calcification, occlusion, and complex lesions. Much more contrast agent is needed during PCI, and combined with renal dysfunction and a high rate of diabetes, the risk of contrast-induced nephropathy is dramatically increased [27], further exacerbating the condition. All the above reasons would lead to more cardiovascular events such as repeated revascularization and cardiac mortality after PCI. The 2018 ESC/EACTS Guidelines on myocardial revascularization recommended CABG for stable angina patients with three-vessel disease and diabetes (Class IA recommendation). Our research conclusions are aligned with this guideline recommendation [10].

A meta-analysis that included 2 RCTs and 15 retrospective experiments reported that contrary to our findings, the incidence of short-term all-cause death of CABG is higher than PCI [21]. At the same time, it is interesting that a summary analysis of two RCTs with short-term MACCEs and repeated revascularization outcomes found that the risk of MACCEs and the proportion of patient participants who underwent PCI who require repeated revascularization were significantly reduced than participants who underwent CABG. Therefore, further research is required to explore whether CKD participants with CAD who undergo PCI or CABG have similar short-term risks.

### Limitations

The novelty of this study is that we included all randomized controlled experiments. However, our research has several restrictions. Firstly, the amount of people participating in the research is still limited. Secondly, the SYNTAX scale tool is a specific instrument introduced by the Synergy between Percutaneous Coronary Intervention with TAXUS and Cardiac Surgery (SYNTAX) trial, which was utilized to assess the severity as well as the progression of CAD. Because of the lack of SYNTAX scale data in the majority of the research involved, the analysis of subgroups according to different SYNTAX score ranges were unfinished. Thirdly, a previous study has shown that patients undergoing peritoneal dialysis have a decreased incidence of hemorrhagic stroke than patients undergoing hemodialysis [28]. Nevertheless, an analysis of subgroups according to different dialysis types (peritoneal dialysis and hemodialysis) couldn’t be implemented because the involved research did not offer enough information on dialysis types. Fourth, many included studies did not evaluate drug therapy, which may affect long-term results. What’s more, our study was the lack of evaluation of patients with renal dysfunction categorized according to eGFR due to the subgroup analyses were not being performed in the five included research. Last but not least, our study obtained a limited sample with a total of 1198 patients, conclusion needs to be further studied in a larger population.

## Conclusion

Thus, in accordance with the long-term follow-up results, CABG is still better than PCI for CAD patients with CKD. However, further large-sample RCT experiments are still needed to confirm the short-term MACCEs risk of PCI and CABG.

## Data Availability

The datasets used and/or analyzed during the current study are available from the corresponding author upon reasonable request.

## Abbreviations

CAD: Coronary artery disease
CKD: Chronic kidney disease
MACCEs: Major cardiovascular and cerebrovascular adverse events
MI: Myocardial infarction
RCT: Randomized control trial

## References

[1] Thompson S, James M, Wiebe N, Hemmelgarn B, Manns B, Klarenbach S (2015). Cause of death in patients with reduced kidney function. J Am Soc Nephrol.

[2] Wang C, Liu J (2006). Research progress of chronic Kidney Disease complicated with coronary artery Disease. Chin J Pract Int Med.

[3] Baber U, Farkouh ME, Arbel Y, Muntner P, Dangas G, Mack MJ (2016). Comparative efficacy of coronary artery bypass Surgery vs. percutaneous coronary intervention in patients with Diabetes and multivessel coronary artery Disease with or without chronic Kidney Disease. Eur Heart J.

[4] Giustino G, Mehran R, Serruys PW, Sabik JF 3rd, Milojevic M, Simonton CA, et al. Left main revascularization with PCI or CABG in patients with chronic Kidney Disease: EXCEL trial. J Am Coll Cardiol. 2018;72(7):754–65. 10.1016/j.jacc.2018.05.057 30092952

[5] Szummer K, Lundman P, Jacobson SH, Schon S, Lindback J, Stenestrand U (2009). Influence of renal function on the effects of early revascularization in non-ST-elevation Myocardial Infarction: data from the Swedish web-system for enhancement and development of evidence-based care in Heart Disease evaluated according to recommended therapies (SWEDEHEART). Circulation.

[6] Huang HD, Alam M, Hamzeh I, Virani S, Deswal A, Aguilar D (2013). Patients with severe chronic Kidney Disease benefit from early revascularization after acute coronary syndrome. Int J Cardiol.

[7] Vuurmans T, Er L, Sirker A, Djurdjev O, Simkus G, Levin A (2018). Long-term patient and kidney survival after coronary artery bypass grafting, percutaneous coronary intervention, or medical therapy for patients with chronic Kidney Disease: a propensity-matched cohort study. Coron Artery Dis.

[8] Milojevic M, Head SJ, Mack MJ, Mohr FW, Morice MC, Dawkins KD (2018). The impact of chronic Kidney Disease on outcomes following percutaneous coronary intervention versus coronary artery bypass grafting in patients with complex coronary artery Disease: five-year follow-up of the SYNTAX trial. EuroIntervention.

[9] Kang SH, Lee CW, Yun SC, Lee PH, Ahn JM, Park DW (2017). Coronary artery bypass grafting vs. drug-eluting stent implantation for Multivessel Disease in patients with chronic Kidney Disease. Korean Circ J.

[10] Neumann FJ, Sousa-Uva M, Ahlsson A, Alfonso F, Banning AP, Benedetto U (2019). 2018 ESC/EACTS guidelines on myocardial revascularization. EuroIntervention.

[11] Arnett DK, Blumenthal RS, Albert MA, Buroker AB, Goldberger ZD, Hahn EJ (2019). 2019 ACC/AHA Guideline on the primary Prevention of Cardiovascular Disease: a report of the American College of Cardiology/American Heart Association Task Force on Clinical Practice guidelines. Circulation.

[12] Park SJ, Ahn JM, Kim YH, Park DW, Yun SC, Lee JY (2015). Trial of everolimus-eluting stents or bypass Surgery for coronary Disease. N Engl J Med.

[13] Bangalore S, Guo Y, Samadashvili Z, Blecker S, Xu J, Hannan EL (2015). Revascularization in patients with Multivessel Coronary Artery Disease and chronic Kidney Disease: Everolimus-Eluting stents Versus Coronary artery bypass graft Surgery. J Am Coll Cardiol.

[14] Nagendrababu V, Dilokthornsakul P, Jinatongthai P, Veettil SK, Pulikkotil SJ, Duncan HF (2020). Glossary for systematic reviews and meta-analyses. Int Endod J.

[15] Sterne JAC, Savovic J, Page MJ, Elbers RG, Blencowe NS, Boutron I (2019). RoB 2: a revised tool for assessing risk of bias in randomised trials. BMJ.

[16] Fourth universal definition of myocardial infarction. (2018). Rev Esp Cardiol (Engl Ed). 2019;72(1):72. 10.1016/j.rec.2018.11.011 30580786

[17] Higgins JP, Altman DG, Gotzsche PC, Juni P, Moher D, Oxman AD (2011). The Cochrane collaboration’s tool for assessing risk of bias in randomised trials. BMJ.

[18] Lima EG, Charytan DM, Hueb W, de Azevedo DFC, Garzillo CL, Favarato D (2020). Long-term outcomes of patients with stable coronary Disease and chronic kidney dysfunction: 10-year follow-up of the Medicine, Angioplasty, or Surgery study II trial. Nephrol Dial Transplant.

[19] Ix JH, Mercado N, Shlipak MG, Lemos PA, Boersma E, Lindeboom W (2005). Association of chronic Kidney Disease with clinical outcomes after coronary revascularization: the arterial revascularization therapies study (ARTS). Am Heart J.

[20] Aoki J, Ong AT, Hoye A, van Herwerden LA, Sousa JE, Jatene A (2005). Five year clinical effect of coronary stenting and coronary artery bypass grafting in renal insufficient patients with multivessel coronary artery Disease: insights from ARTS trial. Eur Heart J.

[21] Wu P, Luo F, Fang Z (2019). Multivessel Coronary revascularization strategies in patients with chronic Kidney Disease: a Meta-analysis. Cardiorenal Med.

[22] Kumada Y, Ishii H, Aoyama T, Kamoi D, Kawamura Y, Sakakibara T (2014). Long-term clinical outcome after surgical or percutaneous coronary revascularization in hemodialysis patients. Circ J.

[23] Doenst T, Haverich A, Serruys P, Bonow RO, Kappetein P, Falk V (2019). PCI and CABG for treating stable coronary artery Disease: JACC Review topic of the Week. J Am Coll Cardiol.

[24] de Boer IH, Rue TC, Hall YN, Heagerty PJ, Weiss NS, Himmelfarb J (2011). Temporal trends in the prevalence of diabetic Kidney Disease in the United States. JAMA.

[25] Hill NR, Fatoba ST, Oke JL, Hirst JA, O’Callaghan CA, Lasserson DS (2016). Global prevalence of chronic Kidney Disease - A systematic review and Meta-analysis. PLoS ONE.

[26] Hanna EB, Chen AY, Roe MT, Wiviott SD, Fox CS, Saucedo JF (2011). Characteristics and in-hospital outcomes of patients with non-ST-segment elevation Myocardial Infarction and chronic Kidney Disease undergoing percutaneous coronary intervention. JACC Cardiovasc Interv.

[27] Silver SA, Shah PM, Chertow GM, Harel S, Wald R, Harel Z (2015). Risk prediction models for contrast induced Nephropathy: systematic review. BMJ.

[28] Zhang Z, Ni H, Xu X (2014). Do the observational studies using propensity score analysis agree with randomized controlled trials in the area of sepsis?. J Crit Care.

